# Efficacy and Safety of Integrated Traditional Chinese Medicine and Biologic Treatment in Moderate to Severe Atopic Dermatitis: Protocol for a Prospective Observational Real-World Study

**DOI:** 10.2196/96547

**Published:** 2026-08-07

**Authors:** Fanlingzi Shen, Ruiqi Cai, Jinrong Lu, Xiangjin Gao, Wencheng Jiang, Bin Li, Ruiping Wang

**Affiliations:** 1 Clinical Research Center, Shanghai Skin Disease Hospital, 1278 Baode Road, Jing’an District, Shanghai, China, 86 (021)36803000; 2 School of Public Health, Shanghai University of Traditional Chinese Medicine, Shanghai, China; 3 Dermatology of Traditional Chinese Medicine, Shanghai Skin Disease Hospital, Shanghai, China

**Keywords:** atopic dermatitis, integrative medicine, traditional Chinese medicine, biologic treatment, real-world study, study protocol

## Abstract

**Background:**

Atopic dermatitis (AD) is a chronic and relapsing skin disorder that significantly impairs patients’ quality of life. Long-term biologic therapy provides durable efficacy for AD, but raises concerns related to costs, safety, and withdrawal-associated relapse. Traditional Chinese medicine (TCM) has a long history in AD treatment and is usually integrated with biologic treatment to achieve complementary benefits and provide comprehensive care for patients with AD. However, high-quality evidence supporting its real-world efficacy remains limited.

**Objective:**

This study aims to collect real-world data to evaluate the efficacy and safety of TCM integrated with biologics (TCMIB) in moderate to severe AD, thereby providing methodological insights to obtain high-quality evidence and inform future in-depth research on AD treatment.

**Methods:**

A total of 3500 patients with moderate to severe AD will be classified into 7 groups based on clinical manifestations and receive 16 weeks of treatment followed by 36 weeks of observation. A reduction of 75% or more in eczema area and severity index score from baseline to week 16 is set as the primary outcome. The secondary outcomes include the eczema area and severity index, body surface area, Investigator’s Global Assessment scale, Dermatology Life Quality Index, numerical rating scale for pruritus, Patient-Oriented Eczema Measure, Atopic Dermatitis Control Tool, and TCM syndrome scale. Assessments will be performed at baseline, every 2 weeks until week 16, and then every 4 weeks until week 52. Safety assessments include vital signs, concomitant medications, and adverse events. The SAS software (version 9.4) will be used for data analysis, and a *P* value of less than .05 will be considered statistically significant.

**Results:**

This study has been approved by the institutional review boards of Shanghai Skin Disease Hospital and registered on the International Traditional Medicine Clinical Trial Registry (ITMCTR2025000984). Patient recruitment began in June 2025 and is expected to be completed in January 2028. Data analysis will begin in June 2028. The main results of the study are expected to be submitted for publication in peer-reviewed journals in December 2028.

**Conclusions:**

The anticipated findings of this protocol are projected to furnish strong evidence on the efficacy and safety of TCMIB for moderate to severe AD, potentially contributing to the standardization of TCMIB and optimization of clinical practice.

## Introduction

### Background

Atopic dermatitis (AD) is a chronic and relapsing inflammatory skin disorder that exhibits distinct clinical manifestations across different age groups [1]. Its key features include an eczematous eruption accompanied by intense itch [2]. AD is commonly associated with other atopic conditions, including food allergy, asthma, and allergic rhinitis [3]. Worldwide, AD affects more than 200 million people, with estimated prevalence rates of 15% to 25% in children and 3% to 7% in adults [2]. According to the 2019 Global Burden of Disease study, there were 35.58 million cases of AD and 1.54 million years lived with disability attributable to AD in China [4]. AD imposes a substantial economic burden due to both direct medical costs and decreased productivity [5]. Furthermore, AD significantly impairs the quality of life of patients. Pruritus, depression, sleep disturbance, and anxiety are the most cited impacts of AD causing clinical and humanistic burden [6].

In China, the clinical management of AD adheres to a stepwise treatment based on disease severity [7]. Fundamental therapy includes regular use of moisturizer and emollient, and avoidance of triggering factors. For mild to moderate AD, topical corticosteroids or topical calcineurin inhibitors can be used according to the patient’s age, lesion characteristics, affected areas, and severity of condition. Oral antihistamines may also be prescribed as needed to alleviate pruritus or manage comorbid allergic symptoms. In cases of moderate to severe AD, systemic treatment options are introduced, including immunosuppressants, short-term systemic corticosteroids, phototherapy, Janus kinase inhibitors, and biologic agents [8,9]. Despite the availability of multiple conventional therapies for AD, their efficacy is often limited, or their safety profile is unsatisfactory in some patients with moderate to severe disease, making it difficult to meet the clinical needs [10].

In recent years, the emergence of biologic agents has transformed the AD treatment landscape, enabling patients to achieve a high level of disease control [11]. Expert consensus indicates that biologic agents can be used as first-line systemic therapy for patients with moderate to severe AD with inadequate response to or who are unsuitable for topical therapies [8]. Dupilumab is the first biologic agent approved for AD, which marked the transition from broad-spectrum systemic immunosuppressants to precision-targeted treatment [12]. By targeting interleukin-4 receptor α, dupilumab inhibits the signaling of both interleukin-4 and interleukin-13, thereby reducing inflammatory responses and alleviating pruritus [11]. Stapokibart is the first domestically developed interleukin-4 receptor α antibody drug approved in China, having received regulatory approval in September 2024 for the treatment of moderate to severe AD in adults [13]. Biologics have significantly improved the capacity to achieve treatment goals for AD. However, their risk-benefit profiles still need to be carefully considered due to the high treatment costs, the requirement for biweekly subcutaneous injections, and the potential for disease relapse following discontinuation [14-17]. Consequently, exploring more diverse and sustainable long-term management strategies has become a critical and urgent issue.

Traditional Chinese medicine (TCM) and integrated traditional Chinese and Western medicine (ICWM) offer novel perspectives and directions for the clinical management of AD, especially for patients with moderate to severe AD. TCM has unique clinical advantages in understanding and treating AD. According to TCM theory, the pathogenesis of AD is attributed to internal factors such as heart fire and spleen dampness, as well as external factors such as wind evil [18]. TCM treatment emphasizes syndrome differentiation and holistic regulation, aiming to rebalance the body by addressing immune dysregulation, restoring skin barrier function, and promoting systemic equilibrium. This approach demonstrates considerable advantages in terms of treatment efficacy, reduction in side effects, and prevention of relapse. ICWM represents a treatment model with distinctive Chinese characteristics. For patients with severe AD, TCM integrated with biologics (TCMIB) can facilitate rapid disease control and alleviate clinical symptoms [14]. Previous studies have suggested that combining TCM with dupilumab may offer superior clinical efficacy compared to dupilumab monotherapy in moderate to severe AD, resulting in better lesion clearance, greater itch reduction, and improved quality of life [19,20]. However, due to limited sample sizes and insufficient evidence, definitive conclusions regarding the efficacy and safety of TCMIB for AD cannot be drawn. Therefore, we will conduct a prospective real-world study on patients with AD undergoing TCMIB treatment.

### Objectives

In this study, we plan to collect clinically relevant data from patients in a real-world setting. The primary objective of this study is to evaluate the efficacy and safety of TCMIB treatment at 16 weeks in patients with moderate to severe AD, and the secondary objective is to analyze data on the involvement of TCM in the diagnosis, treatment, constitutional regulation, and prevention of relapse in AD so as to provide methodological insights to obtain high-quality evidence and inform future in-depth research on AD treatment. The findings are expected to provide further evidence for standardizing TCMIB regimens in the treatment of AD.

## Methods

### Study Design

**Figure 1.**
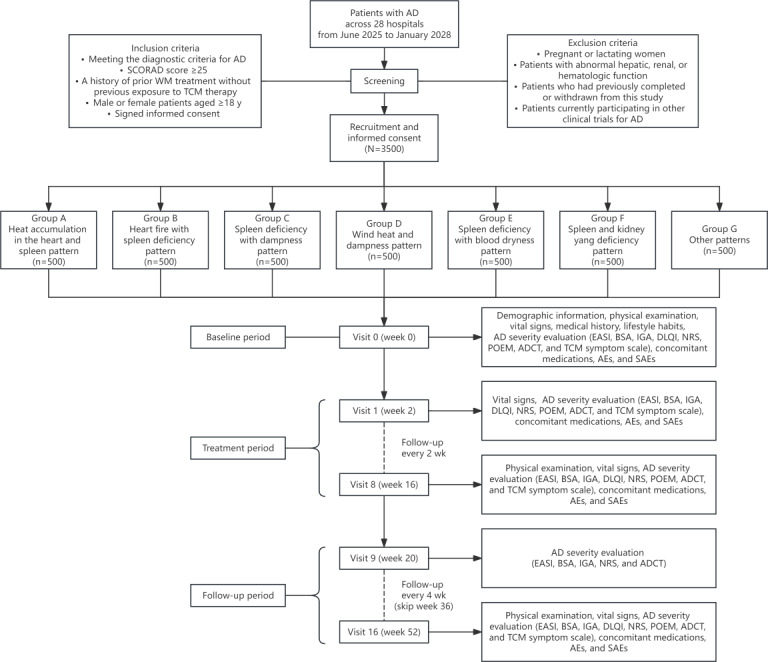
Study flowchart. AD: atopic dermatitis; ADCT: Atopic Dermatitis Control Tool; AE: adverse event; BSA: body surface area; DLQI: Dermatology Life Quality Index; EASI: eczema area and severity index; IGA: Investigator’s Global Assessment scale; NRS: numerical rating scale; POEM: Patient-Oriented Eczema Measure; SAE: serious adverse event; SCORAD: Scoring Atopic Dermatitis; TCM: traditional Chinese medicine; WM: Western medicine.

This is a multicenter, prospective, real-world study that will be conducted at 28 hospitals participating in the National Clinical Research Center for Skin and Immune Diseases Real-World Big Data Collection Platform from October 2024 to September 2028. The hospitals are listed in Table S1 in Multimedia Appendix 1. On the basis of the “disease-syndrome” classification framework, patients will be assigned to 7 groups according to their specific clinical presentations (n=500 per group): heat accumulation in the heart and spleen pattern (group A), heart fire with spleen deficiency pattern (group B), spleen deficiency with dampness pattern (group C), wind heat and dampness pattern (group D), spleen deficiency with blood dryness pattern (group E), spleen and kidney yang deficiency pattern (group F), and other patterns (group G). Patients in all groups will undergo a 16-week treatment phase and then a 36-week follow-up period. Baseline is defined as the time of enrollment (week 0) prior to the initiation of any treatment. Assessments will be conducted at baseline, every 2 weeks until week 16, and then every 4 weeks until week 52. The flowchart of this study is shown in Figure 1.

### Diagnostic Criteria, Inclusion Criteria, and Exclusion Criteria

The diagnostic criteria of AD are based on the Chinese Guideline for the Diagnosis and Treatment of Atopic Dermatitis (2020 edition) [9], and the diagnostic criteria of TCM patterns are based on the guidelines for diagnosis and treatment of AD in TCM (DB44/T 2259-2020) [21], a local standard of Guangdong province.

The inclusion criteria for this study are as follows: (1) meeting the diagnostic criteria for AD, (2) a Scoring Atopic Dermatitis score of 25 or higher, (3) a history of Western medicine treatment but without previous TCM treatment, (4) male or female patients over 18 years of age, and (5) signature of the informed consent form.

The exclusion criteria comprise the following: (1) pregnant or lactating women; (2) patients with abnormal hepatic, renal, or hematologic function; (3) patients who previously completed or withdrew from this study; and (4) those currently participating in other clinical trials for AD.

### Treatment Plan

The treatment plans of patients included in the study are formulated in accordance with the expert consensus on TCM diagnosis and treatment of AD [22] and the guidelines for diagnosis and treatment of AD in TCM [21]. Patients in groups A to F will receive both TCM and biologic treatment (TCMIB groups), whereas patients in group G will receive biologic treatment only. TCM prescriptions include Sanxin Daochi decoction, Peitu Qingxin decoction, Xiao’er Huashi decoction, Shenling Baizhu powder, Xiaofeng powder, Jianpi Runfu decoction, Zhenwu decoction, and Wuling powder. All TCM prescriptions are administered as decoctions for oral use. The standard dosage is 1 dose per day, which is decocted in water and divided into 2 warm servings of 200 mL each. Dermatologists are permitted to individualize the base prescriptions according to each patient’s specific pattern differentiation. The biologic agent used in this study is dupilumab. The dosing regimen follows the expert consensus for dupilumab in the treatment of AD [10]: an initial dose of 600 mg (administered as two 300-mg subcutaneous injections) with a maintenance dose of 300 mg subcutaneously every 2 weeks. Patients in all 7 groups will undergo a 16-week treatment phase and then a 36-week follow-up period. Detailed treatment regimens are provided in Table S2 in Multimedia Appendix 1.

### Outcomes

#### Primary Outcome

The eczema area and severity index (EASI) is one of the most widely used severity scales for assessing AD skin lesions, quantifying the severity of AD based on the area of involvement and the intensity of 4 clinical signs (erythema, edema or papulation, excoriation, and lichenification) in each body region [23]. The EASI score ranges from 0 to 72, with a higher score indicating a more severe disease condition. In this study, achieving an improvement of 75% or more in the EASI at week 16 from baseline (ie, EASI75) is designated as the primary outcome, calculated using the formula [(EASI at baseline – EASI at week *t*)/EASI at baseline] × 100%. The score will be evaluated at baseline, at every visit during the 16-week treatment period, and throughout the 36-week follow-up.

#### Secondary Outcomes

##### Overview

Secondary outcomes include the following assessments evaluated from baseline to week 52: EASI, body surface area (BSA), Investigator’s Global Assessment (IGA), Dermatology Life Quality Index (DLQI), numerical rating scale (NRS) for pruritus, Patient-Oriented Eczema Measure (POEM), Atopic Dermatitis Control Tool (ADCT), and TCM symptom scale. The EASI score, BSA, IGA, NRS, and ADCT scores will be evaluated at baseline and during the treatment and follow-up period. DLQI, POEM, and TCM syndrome scale scores will be assessed at baseline, during the treatment period, and at week 52.

##### Body Surface Area

The BSA is an assessment tool used to estimate the percentage of total BSA affected by skin lesions. Dermatologists estimate BSA using the “rule of nines,” in which body regions are assigned multiples of 9% (9% for the head and neck, 9% for each upper extremity, 18% each for the anterior and posterior trunk, 18% each for the lower extremities, and 1% for the perineum), or the “palm method,” whereby the patient’s own palm approximates 1% of the total BSA. In this study, the BSA score will be evaluated at baseline, every 2 weeks until week 16, and then every 4 weeks until week 52.

##### Investigator’s Global Assessment

The IGA is a single-item, clinician-reported instrument designed to rapidly grade a patient’s overall disease severity at the time of evaluation. It uses a 6-point ordinal scale (0=clear, 1=almost clear, 2=mild disease, 3=moderate disease, 4=severe disease, and 5=very severe disease). The score is based on a composite judgment of the degree of erythema, infiltration, and papulation [24]. This indicator will be assessed at baseline, every 2 weeks until week 16, and then every 4 weeks until week 52.

##### Dermatology Life Quality Index

The DLQI is a validated instrument designed to measure the impact of skin conditions on quality of life over the previous week [25]. It comprises 10 items assessing 6 domains: symptoms and feelings, daily activities, leisure, work and school, personal relationships, and treatment-related problems. Each item is scored on a 4-point ordinal scale (0=“not at all,” 1=“a little,” 2=“a lot,” and 3=“very much”). The total score ranges from 0 to 30 and is calculated by summing all item scores, with higher scores indicating greater impairment in quality of life. In this study, the DLQI will be evaluated at baseline, every 2 weeks until week 16, and at week 52.

##### NRS for Pruritus

The NRS is a single-item, patient-reported instrument designed to quantify itch severity over the previous 24 hours. Patients are asked to select a numerical rating from 0 to 10, where 0 represents “no itch” and 10 represents “the worst imaginable itch” [26]. This study will assess NRS for pruritus at baseline, every 2 weeks until week 16, and then every 4 weeks until week 52.

##### Patient-Oriented Eczema Measure

The POEM is a self-assessed measurement tool used to assess the frequency of 7 symptoms over the previous week: itching, sleep disturbance, bleeding, weeping or oozing, skin cracking, skin flaking, and skin dryness or roughness. Patients record the frequency of each symptom using a 5-point scale (0=no days, 1=1‐2 days, 2=3‐4 days, 3=5‐6 days, and 4=every day). The total POEM score is the sum of all 7 items, ranging from 0 to 28, with higher scores indicating more severe symptoms [27]. In this study, the POEM will be evaluated at baseline, every 2 weeks until week 16, and at week 52.

##### Atopic Dermatitis Control Tool

The ADCT is designed to assess patient-perceived AD control. It consists of 6 items that patients answer based on their experience over the previous week. These items cover multiple core domains of AD control, including the patient’s perception of AD symptoms, itch, brother, sleep, daily activities, and emotions. Each item is scored using a 5-point response scale ranging from 0 (no problem) to 4 (worst). The total ADCT score is calculated by summing the scores of all 6 items, ranging from 0 to 24, with higher scores indicating poorer disease control. In clinical practice, an ADCT score of 7 or higher is considered indicative of inadequately controlled disease [28]. In this study, the ADCT will be evaluated at baseline, every 2 weeks until week 16, and then every 4 weeks until week 52.

##### TCM Symptom Scale

The TCM symptom scale is a nonquantitative assessment instrument consisting of 2 primary components: local symptom evaluation and systemic symptom evaluation. The local assessment focuses on skin lesion characteristics and pruritus severity, whereas the systemic evaluation systematically documents facial complexion, sleep quality, dietary preferences, bowel and urinary habits, tongue coating, and pulse characteristics. The assessments of the TCM syndrome scale will be conducted at baseline, every 2 weeks until week 16, and at week 52.

### Sample Size

The improvement of 75% or more in the eczema area and severity index (EASI75) response rate at week 16 will be adopted as the primary efficacy outcome in this study. On the basis of prior data [29], a 57% response rate will be assumed in patients with biologic treatment (group G; *p*_1_= 0.57), and a 70% response rate will be assumed for the TCMIB group (groups A-F; *p*_2_=0.70). A 2-sided significance level (α) of .01 and a statistical power (1 − β) of 0.90 are specified. According to the sample size calculation formula n = (μ_α/2_ + μ_β_)^2^ × [*p*_1_(1 − *p*_1_) + *p*_2_(1 − *p*_2_)]/(*p*_2_ − *p*_1_)^2^, and considering a 15% dropout rate, at least 472 patients should be enrolled in each group. The final sample size is determined to be 500 patients per group. A total of at least 3500 participants are planned to be enrolled. Additionally, each participating hospital is expected to recruit a minimum of 100 patients, with at least 10 patients in each of the 7 treatment groups.

### Safety Assessment

In this study, safety assessment will encompass monitoring of vital signs, recording of concomitant medications, physical examination, and recording of all adverse events (AEs) and serious AEs (SAEs). Vital signs, concomitant medications, AEs, and SAEs will be monitored at baseline, throughout the treatment period, and at week 52, whereas physical examinations will be performed at baseline, week 16, and week 52. A comprehensive study schedule is provided in Table S3 in Multimedia Appendix 1.

In this study, an AE is defined as any untoward medical occurrence in a patient following the initiation of treatment, which includes the presence or worsening of any syndrome, symptom, or disease. An SAE refers to any event occurring during the study that results in any of the following outcomes: hospitalization, prolongation of existing hospitalization, disability, inability to work, congenital anomaly, life-threatening condition, or death. Throughout the treatment period, the dermatologists will closely monitor the patients’ conditions. When AEs occur, the dermatologists will provide appropriate care based on the clinical presentation and will document relevant details, including the onset date, severity, relationship to study therapy, and resolution date of each event. All AEs will be monitored and followed up on until they are alleviated or properly resolved. Participants with AEs can withdraw from the study immediately and will receive appropriate medical management without cost. All AEs and SAEs will be reported in accordance with relevant regulatory requirements.

### Data Collection and Management

We have designed a standardized case report form (CRF) for data collection. The CRF comprises the following sections: (1) demographic data, including age, gender, and educational level; (2) vital signs and physical examination, including blood pressure, pulse, height, and weight; (3) medical history, including past medical history, comorbidities, family history, and allergy history; (4) lifestyle habits (tobacco smoking and alcohol consumption); (5) AD assessments from baseline to week 52, including EASI, BSA, IGA, DLQI, NRS, POEM, ADCT, and TCM symptom scale; and (6) previous and current medications and AEs.

The aforementioned information will be collected by well-trained and experienced assessors through face-to-face interviews and subsequently documented in the CRFs. All CRFs will be verified by specialized personnel to preserve the integrity and accuracy of the information. CRF data will be uploaded to the National Clinical Research Center for Skin and Immune Diseases Real-World Big Data Collection Platform. Medical records will be retained at each study site in both paper and electronic formats. All data will be anonymized by removing any personally identifiable information that could directly reveal participant identities.

### Statistical Analysis

This study will use the SAS software (version 9.4; SAS Institute) for statistical analysis. The reasons for missing data will be analyzed, and the multiple imputation method will be used for data completion. Statistical description and analysis will be performed on demographic characteristics and efficacy evaluation data. Quantitative variables following a normal distribution will be described using means and SDs and analyzed using either the 2-tailed *t* test or 2-tailed paired *t* test. Skew-distributed quantitative variables will be summarized as medians and IQRs and analyzed using the Wilcoxon rank-sum test. The quantitative variables of repeated measurement will be analyzed via repeated-measure ANOVA. Qualitative variables will be expressed as frequencies and percentages. The comparison between groups for categorical data will be conducted using the chi-square test, whereas repeated-measurement categorical data will be analyzed using the generalized estimating equations method. Generalized estimating equations models will be fitted with achievement of EASI75 response at each visit as the dependent variable and treatment regimen as the independent variable, adjusted for demographic characteristics (age and sex), medical history (family history and comorbidities), baseline disease severity (EASI, BSA, and IGA), and lifestyle factors (smoking and drinking). The working correlation structure will be selected based on the minimum quasi-likelihood under the independence model criterion. In addition, subgroup analyses will be performed stratified by age, sex, and disease severity. In this study, a *P* value below .05 will be considered statistically significant.

### Ethical Considerations

This study protocol has been approved by the institutional review boards of Shanghai Skin Disease Hospital (approval 2024-34) and registered on the International Traditional Medicine Clinical Trial Registry (ITMCTR2025000984). Ethics approval has also been obtained independently from the institutional review boards of all participating hospitals. The study will be conducted in strict compliance with the Declaration of Helsinki, the International Council for Harmonisation of Technical Requirements for Pharmaceuticals for Human Use’s good clinical practice, the National Medical Products Administration’s good clinical practice, and other relevant regulations. Prior to the initiation of any study-related procedures, written informed consent must be obtained from each participant, their legally authorized representative, or an impartial witness.

## Results

This study was reviewed and received ethics approval in September 2024, was funded in January 2025, and was registered in May 2025. Patient recruitment began in June 2025, with 900 patients recruited as of manuscript submission and is scheduled to conclude in January 2028. Data analysis is planned to commence in June 2028, and the primary results are expected to be submitted to a peer-reviewed journal in December 2028.

## Discussion

### Expected Findings

AD is a chronic inflammatory skin disease characterized by intense pruritus and a relapsing-remitting course. Moderate to severe AD often requires systemic therapy, and conventional systemic treatments are ineffective or intolerable for some patients. The introduction of novel systemic therapies, including biologics and Janus kinase inhibitors, has reshaped the treatment strategy for AD. Dupilumab is one of the most widely used biologic agents for AD in clinical practice in China, demonstrating significant efficacy in relieving pruritus, improving skin lesions, and enhancing quality of life [30]. Nevertheless, some patients still fail to achieve adequate disease control, creating a pressing need for alternative treatment approaches.

Complementary and alternative medicine is increasingly being used in the management of AD. TCM, as a major component of complementary and alternative medicine, has demonstrated unique value in the treatment of AD. The application of TCM for AD has a long history. In TCM theory, AD is designated as “wind of four fossae” (Si Wan Feng). The clinical presentation of this disorder, as documented in the seminal TCM canon *Golden Mirror of Medicine* (Yi Zong Jin Jian), closely correlates with contemporary Western medical descriptions of AD [14]. Chinese herbal medicines and their formulations exert pleiotropic effects through multiple components, pathways, and targets. They can alleviate and treat AD via various mechanisms, thereby playing a significant role in its management [18]. Previous systematic reviews and meta-analyses have indicated that Chinese herbal medicine can effectively improve the severity of AD symptoms [31]. The ICWM regimen represents an effective clinical strategy for AD management, leveraging the strengths of both systems to compensate for their respective limitations. Previous studies have demonstrated that the combination of dupilumab with TCM formulas or proprietary Chinese medicines, such as Shenling Baizhu powder [20] plus or minus Jianpi Chushi decoction [19] and Runzao Zhiyang capsule [32], can enhance clinical efficacy in patients with moderate to severe AD. The overall response rate with the combination therapy has been shown to be superior to that of dupilumab monotherapy. However, these conclusions warrant further verification through higher-level evidence-based clinical studies. Although traditional randomized controlled trials are considered the gold standard for evaluating clinical efficacy, their stringent inclusion and exclusion criteria and standardized interventions often exclude various influencing factors present in real-world clinical settings, which limits the generalizability of the findings. Therefore, there is a compelling need to use real-world studies with real-world data to analyze the effectiveness and safety of the TCMIB regimen for AD.

This protocol outlines the design of a prospective, multicenter, large-scale observational study that provides a real-world setting to explore the role of TCM in the diagnosis and treatment of, constitutional regulation of, and prevention of relapse in AD. To our knowledge, this is the first multicenter observational study in China to enroll at least 3500 patients with moderate to severe AD. The study will analyze the demographic and TCM-specific characteristic profiles of the AD cohort and identify optimal TCMIB therapeutic strategies through long-term observation. Furthermore, it will provide critical data on the effectiveness and safety of a 16-week TCMIB intervention and explore whether this treatment yields additional clinical benefits. Beyond these immediate goals, the creation of a shared clinical database is expected to provide substantial data support for subsequent TCM research, ultimately contributing to the progression of AD clinical research and management.

There are some limitations to this study. First, patient-reported outcomes such as NRS for pruritus and POEM are inherently subjective and may be biased by interindividual differences in symptom perception thresholds. Second, the total period of this study is 52 weeks (including a 16-week treatment period and a 36-week follow-up period). For various reasons, some patients may have difficulty completing the whole study cycle, resulting in loss to follow-up. Third, as this is a real-world study, patients’ treatment regimens are not randomly assigned, making the results susceptible to certain biases. The potential influence of unmeasured confounding factors on the study results cannot be excluded, which poses challenges to causal inference. Fourth, while external TCM treatment methods (eg, topical Chinese medicine and acupuncture) have all shown therapeutic promise in AD [33-35], this study is restricted to oral TCM formulations. This focus limits the comprehensiveness of the assessment regarding the full therapeutic potential of TCM for AD.

### Conclusions

This study will prospectively collect real-world clinical data from patients with AD receiving TCMIB treatment, thereby establishing a high-quality, multicenter, long-term clinical database in AD management and generating further evidence regarding the clinical effectiveness and safety of TCMIB. The findings are expected to provide a research foundation and inform clinical decision-making for standardizing TCMIB in AD treatment. The study has important significance in realizing the clinical transformation of high-quality real-world medicine evidence and promoting the development of clinical research and practice of AD.

## Supplementary material

10.2196/96547 Multimedia Appendix 1 The hospitals involved in a real-world study evaluating the efficacy and safety of traditional Chinese medicine (TCM) integrated with biologics in moderate to severe atopic dermatitis (AD); basic treatment principles, treatment regimens, and dosage for patients with AD with different TCM pattern types; and timeline for the study.

## Abbreviations

AD: atopic dermatitis
ADCT: Atopic Dermatitis Control Tool
AE: adverse event
BSA: body surface area
CRF: case report form
DLQI: Dermatology Life Quality Index
EASI: eczema area and severity index
EASI75: improvement of 75% or more in the eczema area and severity index
ICWM: integrated traditional Chinese and Western medicine
IGA: Investigator’s Global Assessment
NRS: numerical rating scale
POEM: Patient-Oriented Eczema Measure
SAE: serious adverse event
TCM: traditional Chinese medicine
TCMIB: traditional Chinese medicine integrated with biologics
