# Supplementary material for: Efficacy and Safety of Integrated Traditional Chinese Medicine and Biologic Treatment in Moderate to Severe Atopic Dermatitis: Protocol for a Prospective Observational Real-World Study
Source: JMIR Res Protoc. 2026 Aug 7;15:e96547. doi: 10.2196/96547 (PMC13454249; doi:10.2196/96547)
Supplement: Supplementary file 1 [file resprot-v15-e96547-s001.docx]

Table S1. The hospitals involved in a real-word study evaluating the efficacy and safety of TCMIB in moderate-to-severe AD

| No. | Province | Hospital |
| --- | --- | --- |
| 1 | Anhui | The First Affiliated Hospital of Anhui University of Traditional Chinese Medicine |
| 2 | Beijing | Beijing Traditional Chinese Medicine Hospital Affiliated to Beijing University of Traditional Chinese Medicine |
| 3 | Beijing | Guang'anmen Hospital, Chinese Academy of Traditional Chinese Medicine |
| 4 | Beijing | Oriental Hospital Affiliated to Beijing University of Traditional Chinese Medicine |
| 5 | Chongqing | Chongqing Hospital of Traditional Chinese Medicine |
| 6 | Guangdong | Guangdong Provincial Hospital of Traditional Chinese Medicine |
| 7 | Guangdong | Shenzhen Hospital of Traditional Chinese Medicine |
| 8 | Guizhou | The First Affiliated Hospital of Guizhou University of Traditional Chinese Medicine |
| 9 | Heilongjiang | Qiqihar Traditional Chinese Medicine Hospital |
| 10 | Henan | The First Affiliated Hospital of Henan University of Traditional Chinese Medicine |
| 11 | Hubei | Hubei Provincial Hospital of Traditional Chinese Medicine |
| 12 | Hubei | Wuhan first Hospital |
| 13 | Hunan | The Second Affiliated Hospital of Hunan University of Traditional Chinese Medicine |
| 14 | Jiangsu | Jiangyin Hospital of Traditional Chinese Medicine |
| 15 | Jiangsu | Suzhou Traditional Chinese Medicine Hospital |
| 16 | Jiangxi | Jiangxi Provincial Hospital of Traditional Chinese Medicine |
| 17 | Liaoning | Shenyang Seventh People's Hospital |
| 18 | Shanghai | Longhua Hospital Affiliated to Shanghai University of Traditional Chinese Medicine |
| 19 | Shanghai | Shanghai Skin Diseases Hospital |
| 20 | Shanghai | Yueyang Hospital of Integrated Traditional Chinese and Western Medicine. Shanghai University of Traditional Chinese Medicine |
| 21 | Shanxi | Shaanxi Provincial Hospital of Traditional Chinese Medicine |
| 22 | Sichuan | Affiliated Hospital of Chengdu University of Traditional Chinese Medicine |
| 23 | Tianjin | Affiliated Hospital of Tianjin Academy of Traditional Chinese Medicine |
| 24 | Xinjiang | Xinjiang Uygur Autonomous Region Traditional Chinese Medicine Hospital |
| 25 | Zhejiang | Hangzhou Hospital of Traditional Chinese Medicine |
| 26 | Zhejiang | Hangzhou Third People's Hospital |
| 27 | Zhejiang | Ningbo Hospital of Traditional Chinese Medicine |
| 28 | Zhejiang | Zhejiang Provincial Hospital of Traditional Chinese Medicine |

AD: Atopic Dermatitis; TCMIB: Traditional Chinese Medicine Integrated with Biologics

Table S2. Basic treatment principles, treatment regimens, and dosage for AD patients with different TCM pattern types

| **Group** | **TCM pattern** | Basic **TCM Treatment Principle** | Treatment Regimen | Dosage and Administration |
| --- | --- | --- | --- | --- |
| A | Heat accumulation in the heart and spleen | Clear heat in the heart and spleen | **Sanxin Daochi** Decoction^a^  + BT**^i^** | All the TCM formulations are to be **decocted in water** for oral administration. The standard dosage is **one dose per day**, divided into **two warm servings of 200 ml each**. The dosage regimen for BT is an initial dose of 600 mg (two 300mg subcutaneous injections), followed by a maintenance dose of 300 mg subcutaneously every 2 weeks. |
| B | Heart fire with spleen deficiency | Clear heart fire and strengthen the spleen | **Peitu Qingxin** Decoction^b^ + BT**^i^** |  |
| C | Spleen deficiency with dampness | Strengthen the spleen and drain dampness | **Xiao'er Huashi** Decoction^c^ + BT**^i^**  **Shenling Baizhu Powder^d^** + BT**^i^** |  |
| D | Wind heat and dampness | Remove wind, clear heat and eliminate dampness | **Xiaofeng Powder^e^** + BT**^i^** |  |
| E | Spleen deficiency with blood dryness | Tonify qi and strengthen the spleen, nourish blood and moisten dryness | Jianpi Runfu Decoction^f^ + BT**^i^** |  |
| F | Spleen and kidney yang deficiency | Strengthen the spleen and induce urination, warm yang and tonify kidney | Zhenwu Decoction^g^ + BT**^i^**  **Wuling** **Powder^h^** + BT**^i^** |  |
| G | Other | **Not applicable** | **BT^i^** |  |

AD: Atopic Dermatitis; BT: Biologic Treatment; TCM: Traditional Chinese Medicine

^a^ Sanxin Daochi Decoction: Forsythiae Fructus 3 g, Gardeniae Fructus 3 g, Nelumbinis Plumula 3 g, Scrophulariae Radix 3 g, Rehmanniae Radix 5 g, Plantaginis Semen 5 g, Cicadae Periostracum 3 g, Junci Medulla 3 g, Glycyrrhizae Radix et Rhizomat 3 g, Poria 5 g.

^b^ Peitu Qingxin Decoction: Lophatheri herba 10 g, Forsythiae fructus 10 g, Junci Medulla 10 g, Rehmanniae Radix 10 g, Atractylodis Macrocephalae Rhizoma 10 g,	Dioscoreae Rhizoma 15 g, Coicis Semen 15 g, Uncariae Ramulus cum Uncis 10 g, Ostreae Concha 15 g, Saposhnikoviae Radix 10 g, Glycyrrhizae Radix et Rhizomat 5 g.

^c^ Xiao'er Huashi Decoction: Atractylodis Rhizoma 10 g, Poria 10 g, Hordei Fructus Germinatus 10 g, Citri Reticulatae Pericarpium 3 g, Alismatis Rhizoma 10 g, Talcum 10 g, Glycyrrhizae Radix et Rhizoma 3 g, Atractylodis Macrocephalae Rhizoma 10 g, Coicis Semen 10 g.

^d^ Shenling Baizhu Powder：Ginseng Radix et Rhizoma 15 g，Poria 15 g，Atractylodis Macrocephalae Rhizoma 15 g，Dioscoreae Rhizoma 15 g，Lablab Semen Album 12 g，Nelumbinis Semen 9 g，Coicis Semen 9 g，Amomi Fructus 6 g，Platycodonis Radix 6 g，Glycyrrhizae Radix et Rhizomat 9 g

^e^ Xiaofeng Powder: Schizonepetae Herba 6 g, Sophorae Flavescentis Radix 6 g, Anemarrhenae Rhizoma 6 g, Atractylodis Rhizoma 6 g, Cicadae Periostracum 6 g, Saposhnikoviae Radix 6 g, Arctii Fructus 6 g, Rehmanniae Radix 6 g, Semen Sesami Nigrum 6 g, Gypsum Fibrosum 6 g, Angelicae Sinensis Radix 6 g, Glycyrrhizae Radix et Rhizoma 3 g, Akebiae Caulis 3 g.

^f^ Jianpi Runfu Decoction: Codonopsis Radix 10 g, Poria 10 g, Atractylodis Rhizoma 10 g, Atractylodis Macrocephalae Rhizoma 10 g, Angelicae Sinensis Radix 10 g, Paeoniae Radix Rubra 10 g, Paeoniae Radix Alba 10 g, Salviae Miltiorrhizae Radix et Rhizomat 10 g,Rehmanniae Radix 15 g, Spatholobi Caulis 15 g, Citri Reticulatae Pericarpium 6 g.

^g^ Zhenwu Decoction: Poria 9 g, Paeoniae Radix Alba 9 g, Atractylodis Macrocephalae Rhizoma 6 g,Zingiberis Rhizoma Recens 9 g, Aconiti Lateralis Radix Praeparata 9 g.

^h^ Wuling Powder: Alismatis Rhizoma 15 g, Polyporus 9 g, Poria 9 g, Atractylodis Macrocephalae Rhizoma 9 g, Cinnamomi Ramulus 6 g.

**^i^** Biologic Treatment: dupilumab

| Events | |  | Baseline |  | Treatment period | | | | | | | |  | Follow-up period | | | | | | | |
| --- | --- | --- | --- | --- | --- | --- | --- | --- | --- | --- | --- | --- | --- | --- | --- | --- | --- | --- | --- | --- | --- |
| Timepoints (week) | |  | 0 |  | 2 | 4 | 6 | 8 | 10 | 12 | 14 | 16 |  | 20 | 24 | 28 | 32 | 40 | 44 | 48 | 52 |
| Patient Enrollment | Inclusion and exclusion criteria |  | ● |  |  |  |  |  |  |  |  |  |  |  |  |  |  |  |  |  |  |
|  | Informed consent |  | ● |  |  |  |  |  |  |  |  |  |  |  |  |  |  |  |  |  |  |
|  | Demographic characteristics |  | ● |  |  |  |  |  |  |  |  |  |  |  |  |  |  |  |  |  |  |
|  | Medical history |  | ● |  |  |  |  |  |  |  |  |  |  |  |  |  |  |  |  |  |  |
|  | Registration |  | ● |  |  |  |  |  |  |  |  |  |  |  |  |  |  |  |  |  |  |
|  | Photographic documentation of lesions |  | ● |  | ● | ● | ● | ● | ● | ● | ● | ● |  | ● | ● | ● | ● | ● | ● | ● | ● |
| Treatment and observation | Groupwise treatment by regimen (Group A-G) |  | ● |  | ● |  |  |  |  |  |  | ● |  |  |  |  |  |  |  |  |  |
| Efficacy evaluation | EASI |  | ● |  | ● | ● | ● | ● | ● | ● | ● | ● |  | ● | ● | ● | ● | ● | ● | ● | ● |
|  | BSA |  | ● |  | ● | ● | ● | ● | ● | ● | ● | ● |  | ● | ● | ● | ● | ● | ● | ● | ● |
|  | IGA |  | ● |  | ● | ● | ● | ● | ● | ● | ● | ● |  | ● | ● | ● | ● | ● | ● | ● | ● |
|  | DLQI |  | ● |  | ● | ● | ● | ● | ● | ● | ● | ● |  |  |  |  |  |  |  |  | ● |
|  | NRS |  | ● |  | ● | ● | ● | ● | ● | ● | ● | ● |  | ● | ● | ● | ● | ● | ● | ● | ● |
|  | POEM |  | ● |  | ● | ● | ● | ● | ● | ● | ● | ● |  |  |  |  |  |  |  |  | ● |
|  | ADCT |  | ● |  | ● | ● | ● | ● | ● | ● | ● | ● |  | ● | ● | ● | ● | ● | ● | ● | ● |
|  | TCM symptom scale |  | ● |  | ● | ● | ● | ● | ● | ● | ● | ● |  |  |  |  |  |  |  |  | ● |
| Safety evaluation | Vital signs |  | ● |  | ● | ● | ● | ● | ● | ● | ● | ● |  |  |  |  |  |  |  |  | ● |
|  | Concomitant medications |  | ● |  | ● | ● | ● | ● | ● | ● | ● | ● |  |  |  |  |  |  |  |  | ● |
|  | Physical examination |  | ● |  |  |  |  |  |  |  |  | ● |  |  |  |  |  |  |  |  | ● |
|  | AEs |  | ● |  | ● | ● | ● | ● | ● | ● | ● | ● |  |  |  |  |  |  |  |  | ● |
|  | SAEs |  | ● |  | ● | ● | ● | ● | ● | ● | ● | ● |  |  |  |  |  |  |  |  | ● |

Table S3. Timeline for a real-word study evaluating the efficacy and safety of TCMIB in moderate-to-severe AD

Group A: heat accumulation in the heart and spleen pattern; Group B: heart fire with spleen deficiency pattern; Group C: spleen deficiency with dampness pattern; Group D: wind heat and dampness pattern; Group E: spleen deficiency with blood dryness pattern; Group F: Spleen and kidney yang deficiency pattern; Group G: other patterns group

AD: Atopic Dermatitis; AE: Adverse Event; SAE: Serious Adverse Event; BSA: Body Surface Area; DLQI: Dermatology Life Quality Index; EASI: Eczema Area and Severity Index; IGA: Investigator’s Global Assessment; NRS: Numerical Rating Scale; POEM: Patient-Oriented Eczema Measure; TCM: Traditional Chinese Medicine; TCMIB: Traditional Chinese Medicine Integrated with Biologics
